# Integrative profiling analysis reveals prognostic significance, molecular characteristics, and tumor immunity of angiogenesis-related genes in soft tissue sarcoma

**DOI:** 10.3389/fimmu.2023.1178436

**Published:** 2023-06-12

**Authors:** Binfeng Liu, Chenbei Li, Chengyao Feng, Hua Wang, Haixia Zhang, Chao Tu, Shasha He, Zhihong Li

**Affiliations:** ^1^ Department of Orthopaedics, The Second Xiangya Hospital of Central South University, Changsha, Hunan, China; ^2^ Hunan Key Laboratory of Tumor Models and Individualized Medicine, The Second Xiangya Hospital of Central South University, Changsha, Hunan, China; ^3^ Department of Oncology, The Second Xiangya Hospital of Central South University, Changsha, Hunan, China

**Keywords:** soft tissue sarcoma, angiogenesis, prognosis, immune landscape, immunotherapy

## Abstract

**Background:**

Soft tissue sarcoma (STS) is a class of malignant tumors originating from mesenchymal stroma with a poor prognosis. Accumulating evidence has proved that angiogenesis is an essential hallmark of tumors. Nevertheless, there is a paucity of comprehensive research exploring the association of angiogenesis-related genes (ARGs) with STS.

**Methods:**

The ARGs were extracted from previous literature, and the differentially expressed ARGs were screened for subsequent analysis. Next, the least absolute shrinkage and selection operator (LASSO) and Cox regression analyses were conducted to establish the angiogenesis-related signature (ARSig). The predictive performance of the novel ARSig was confirmed using internal and external validation, subgroup survival, and independent analysis. Additionally, the association of the ARSig with the tumor immune microenvironment, tumor mutational burden (TMB), and therapeutic response in STS were further investigated. Notably, we finally conducted *in vitro* experiments to verify the findings from the bioinformatics analysis.

**Results:**

A novel ARSig is successfully constructed and validated. The STS with a lower ARSig risk score in the training cohort has an improved prognosis. Also, consistent results were observed in the internal and external cohorts. The receiver operating characteristic (ROC) curve, subgroup survival, and independent analysis further indicate that the novel ARSig is a promising independent prognostic predictor for STS. Furthermore, it is proved that the novel ARSig is relevant to the immune landscape, TMB, immunotherapy, and chemotherapy sensitivity in STS. Encouragingly, we also validate that the signature ARGs are significantly dysregulated in STS, and ARDB2 and SRPK1 are closely connected with the malignant progress of STS cells.

**Conclusion:**

In sum, we construct a novel ARSig for STS, which could act as a promising prognostic factor for STS and give a strategy for future clinical decisions, immune landscape, and personalized treatment of STS.

## Background

Sarcomas are a class of malignant tumors originating from mesenchymal tissue, about 80% of which originate from soft tissue and 20% from bone (1). Among them, soft tissue sarcoma (STS) comprises more than 70 histological subtypes, and the most frequently observed subtypes are leiomyosarcoma, liposarcoma, synovial sarcoma, and rhabdomyosarcoma (2). Although STS is relatively rare, it has a high lethality. According to statistics, more than 5,800 sarcoma patients die yearly in the United States, accounting for 40% of new cases (3). Since it the highly aggressive with early relapse and metastasis, the clinical outcome of STS is not ideal (4). Previous studies have demonstrated that the 5-year survival rate after diagnosis of STS is only 55.5-56.5%, and the patients with metastasis or recurrence are only about 20% (3, 5). Overall, the prognosis of the patient with STS remains dismal, and the development in recent years seems to have gotten stuck in a bottleneck. Therefore, it is urgent to find reliable biomarkers for early diagnosis, risk stratification, and prognosis prediction of STS.

Angiogenesis is the process of forming new blood vessels from pre-existing ones, which offers an adequate metabolic supply and nutrients for tumor growth and is widely considered to play an essential role in tumorigenesis and development (6). With the sustained rapid cellular proliferation and a high metabolic rate of tumor cells, the rapid development of new vascular networks is often required, which is driven by angiogenic factors such as the vascular endothelial growth factor (VEGF) family, hypoxia-inducible factors (HIFs), and fibroblast growth factors (FGFs) (7). Tumor angiogenesis not only supplies nutrients and natural migration pathways for tumors but also promotes tumor progression and regulates the tumor microenvironment (8). Accordingly, targeted tumor angiogenesis therapy has been investigated as a potential anti-tumor therapeutic approach. For instance, anlotinib, a multikinase angiogenesis inhibitor, shows an anti-tumor ability in several STS entities (9). In addition, the identification of promising angiogenesis-related markers and signatures has also been pursued as an attractive strategy for tumor diagnosis and prognostic evaluation. Yuan Yang et al. established a prognosis signature rely on angiogenesis-related genes (ARGs), which can help to predict prognosis, immune infiltration status, and chemotherapy sensitivity in hepatocellular carcinoma (10). However, it remains unclear whether angiogenesis-related signatures (ARSig) can be used in the prognosis and therapy prediction of STS.

Herein, we first constructed a novel signature for STS based on the ARGs, which exhibited excellent predicted performance for the prognosis of STS. Subsequently, the functional enrichment analysis was conducted to investigate the underlying mechanisms. Additionally, the relationships between the ARSig and the tumor immune microenvironment, immune therapy response, and the sensitivity of chemotherapeutic agents were investigated using a serial bioinformatic analysis. It may provide a promising predictor for prognosis prediction and clinical management of STS.

## Methods

### Data collection

The expression profile, copy number variation (CNV), somatic mutation, and clinical characteristics of the STS cohort were downloaded from The Cancer Genome Atlas database (TCGA, https://www.cancer.gov/aboutnci/organization/ccg/research/structural-genomics/tcga). The individual lacking survival information and other clinicopathological features were excluded from subsequent analysis, and the R package “GeoTcgaData” was utilized to convert ensemble ids to gene symbols. In addition, the expression and clinical data of the three independent cohorts (GSE17674, GSE21050, and GSE71118) were extracted from the Gene Expression Omnibus (GEO, https://www.ncbi.nlm.nih.gov/geo/) database. The clinical information of the above patients is shown in **Tables S1-S3**. The R package “AnnoProbe” was applied to map probes, and the R package “limma” was applied to calculate the average values of multiple probes. Among them, the GSE17674 gene set was utilized to identify differentially expressed ARGs, while GSE21050 and GSE71118 cohorts were considered external validation cohorts for the validation analysis. For normalization, the RNA-sequencing data was converted by log2. The ARGs were obtained from previous literature, and their detailed information is shown in **Table S4** (11, 12).

### Identification of differentially expressed ARGs in STS

The R package “limma” was utilized to screen the differential expressed gene with |log2FC| ≥1 and false discovery rate-adjusted P-value ≤ 0.05 (13, 14). Then, the Venn graph was used to confirm the DEARGs. The visualization used the volcano plots and heatmaps based on the R package “ggplot2” and “heat map.” The principal component analysis (PCA) was performed to explore the distribution differences of samples.

### Screening of DEARGs related to the prognosis of STS

To explore the relevance between the DEARGs and the prognosis of STS, we applied the univariate COX regression analysis screening the DEARGs related to prognosis in STS. The screen criteria were set as P-values < 0.05, and these prognostic DEARGs were selected for subsequent signature construction.

### Derivation of angiogenesis-related signatures

All TCGA-STS cohorts (n=260) were randomly split into the training cohort (n=130) and testing (n=130) cohort by “caret” package in R software. In the training cohort, the least absolute shrinkage and selection operator (LASSO) regression analysis was performed to identify candidate signature ARGs from the prognostic DEARGs. Subsequently, the candidate signature ARGs were included in the multivariate Cox regression analysis to construct the optimal ARSig. The ARSig risk score of each STS individual was computed as the following: ARSig risk score = β_i_*X_i_ (β_i_ and X_i_ represent the regression coefficients and expression level of gene i, respectively). Next, every STS cohort was divided into high- and low-risk groups according to the median risk score of the training cohort. To compare the difference in the overall survival (OS) between the distinct ARSig risk groups, we then performed Kaplan-Meier (KM) survival analysis using the “survival” package. In addition, the receiver operating characteristic (ROC) curve and the area under the curve (AUC) were used to assess the predictive accuracy of the novel ARSig (15). The distribution of ARSig risk score and survival status were plotted in R software.

### Evaluation and validation of the novel ARSig

To estimate the credibility of the novel ARSig, we performed the internal and external validation based on the training cohort, the entire cohort, GSE21050, and GSE71118. The above analyses were also conducted in the internal and external validation cohorts. Moreover, the subgroup clinical survival analysis based on different clinical features was performed to investigate the general applicability of the novel ARSig. To assess whether the novel ARSig was an independent indicator of OS in STS, we performed univariate and multivariate Cox regression analyses by combining multiple clinical characteristics. In addition, prognostic signatures for STS based on gene expression were systematically searched from PubMed for predictive performance comparison. **Table S5** includes previously published prognostic models collected in this study.

### Identification of DEGs and functional enrichment analysis

We performed differential expression analysis and functional enrichment analysis to explore the difference in molecular function between the distinct risk groups. Initially, the differentially expressed genes (DEGs) were screened using the limma package. The criterion for screening DEGs was false discovery rate-adjusted P-value < 0.05 and | logFC | > 0.585. Also, the volcano and heat map was applied to visualize the differential expression analysis results. Subsequently, the functional enrichment analysis based on these DEGs was performed utilizing the “clusterProfiler” package, including Gene Ontology (GO) and Kyoto Encyclopedia of Genes and Genomes (KEGG) analysis (16). The functional enrichment analysis results were visualized using the bubble plot.

### Identification of top ten hub genes

The “GOSemSim” package was used to conduct the Friend analysis for screening the hub gene (17). The association between the signature ARGs and each hub gene was investigated utilizing Pearson’s correlation analysis. Then, the difference in the expression of these hub genes between the low- and high-risk groups was compared. The KM survival analysis was applied to explore the relationship between the expression of each hub gene and the OS of patients with STS.

### Gene set enrichment analysis and Gene set variation analysis

To identify the enriched cellular pathways in the high- and low-risk STS cohort, we performed GSEA and GSVA analyses (18, 19). For GSEA, the KEGG gene set (c2.cp.kegg.v7.4.symbols.gmt) was extracted from The Molecular Signatures Database. Then, the GSEA was carried out using the “clusterProfiler” package, and the result was visualized using the R software. Meanwhile, the R package “GSVA” was applied to conduct GSVA analysis, and the limma package was employed to compare the difference in the enriched pathways between the low- and high-risk groups. The pathways with |logFC| > 0.15 and false discovery rate-adjusted P-value < 0.05 were considered significantly enriched pathways and illustrated in clustered heat maps.

### Relationship of ARSig with Tumor Microenvironment, immune checkpoints, and immune cell infiltration in STS

Besides, the association of the novel ARSig with TME and Immune Cell Infiltration was explored in our study. First, we assessed the TME score using the ESTIMATE (Estimation of Stromal and Immune cells in Malignant Tumor tissues using Expression data) algorithm (20). The TME score consists of immune, stromal, and tumor purity scores. Then, the CIBERSORT algorithm was utilized to assess the abundance of immune infiltrating cells (21). Generally, the immune checkpoint gene expression is closely associated with the sensitivity of immunotherapy. Therefore, we obtained the immune checkpoints from previous literature and compared their expression level between the distinct risk groups. Furthermore, the connection between the TME score and immune cell infiltration with the prognosis of STS was investigated by KM survival analysis.

### Mutation and CNV analysis

To explore the relationships between the ARSig and somatic mutations, we analyzed mutation annotation data from the TCGA database using the “maftools” package. Next, the tumor mutation burden (TMB) scores for each STS patient were calculated, and the difference in the TMB scores between the two risk groups was compared by statistical analysis. In addition, the mutations of the genes with mutation Top 20 in the low- and high-risk groups were visualized using waterfall plots. Furthermore, we analyzed the association of the ARSig risk scores with the cancer stem cell (CSC) index.

### Immunotherapy response and drug sensitivity analysis

To further guide the treatment selection for STS, we assess the responses to immunotherapy and chemotherapeutic agent in STS. The response to immunotherapy inhibitors (anti‐CTAL‐4 and anti‐PD‐L1) of STS patients in the distinct risk groups was evaluated by the Subclass Mapping (SubMap) algorithm (22). The Bonferroni correction was employed to correct the P-value of the test level, and the Bonferroni P-value less than 0.05 was considered a statistical significance. For chemotherapy drug sensitivity comparison, the R package “pRRophetic” was applied to determine the half maximal inhibitory concentration (IC50) (23). Then, the Wilcoxon sign-rank test was applied to compare the IC50 of chemotherapy agents between the two different risk groups.

### Establishment of a predictive nomogram

Based on the multivariate Cox progression analysis result, a nomogram composed of independent prognostic factors was constructed using the R package “rms.” (24). Additionally, the calibration curve and decision curve analysis (DCA) draws utilizing the R packages “caret” and “rmda”, which could assess the predictive reliability of the nomogram. Moreover, we further conducted the ROC curve to estimate the predictive performance of the nomogram by using the “survival ROC” package in R software.

### Cell lines and cell culture

The sources of the cell lines used in the present study were all described in previous research (25). All the cell lines were cultured in Dulbecco’s modified Eagle’s medium (DMEM, Procell) containing 10% fetal bovine serum and 1% penicillin-streptomycin solution. Cell cultures were performed at 37°C in a humidified atmosphere containing 5% CO2.

### Quantitative reverse transcription PCR

Total RNA was collected using RNA Express Total RNA Kit (New Cell & Molecular Biotech), and RNA was reverse transcribed utilizing the Revert Aid First Strand cDNA Synthesis Kit (Thermo Scientific), according to the manufacturer’s instructions. Next, RT-qPCR was performed by Hieff qPCR SYBR Green Master Mix (High Rox Plus) (YEASEN Biotech Co., Ltd). The GAPDH was applied for the internal reference for normalization. The relative expression of each gene was calculated with the 2^-ΔΔCT^ method. The specific primer sequences used in the present study are shown in **Table S6**.

### Cell transfection

Negative control (NC), ADRB2, and SRPK1 siRNAs were purchased from Hanbio (Shanghai, China). SW872 cells were seeded in a 6-well plate. When cell area reached 50%, 50nmol NC, ADRB2, and SRPK1 siRNAs were separately transfected into cells using 5uL Lipofectamine 2000 reagent (Invitrogen) for 12 hours. The sequence of siRNA used in our research is illustrated in **Table S7**.

### Cell proliferation assays

Cell counting kit-8 (CCK-8, New Cell & Molecular Biotech) was used to detect the viability of SW872 cells. SW872 cells were placed in a 96-well plate (2000 cells per well) and incubated overnight. Cells were transfected and cultured for indicated times (0, 24, 48, 72, and 96 hours). In each well was added 10ul CCK-8 solution combining 90ul DMEM containing 10% FBS. After 1.5 hours of incubation, the optical absorbance at 450nm was measured with a microplate reader.

### 5-Ethynyl-2’-Deoxyuridine assays

EdU assays (RiboBio) were performed to determine cell proliferation. After transfection, SW872 cells were seeded in 14 ul slippers in 12-well plates. After 48 hours of incubation, cells were cultured using 50um EdU reagent (diluted with DMEM containing 10% FBS at 1:1000) for 2 hours at 37°C. Then, fixed with 4% paraformaldehyde (PFA) and stained with Hoechst solution (diluted with DMEM containing 10% FBS at 1:100).

### Colony-forming assays

The colony-forming assays were carried out for cell proliferation detection. After transfection, 1000 SW872 cells were seeded in 6-well plates and cultured for 2 weeks. Cells were fixed in 4% PFA for 15 minutes and stained with 0.2% crystal violet for 15 minutes.

### Wound healing assay

Wound healing assays were performed to reveal the migration capacity. SW872 cells were placed in different 6-well plates and underwent transfection when the cell area reached 70%. When cell confluence reached 100%, wound healing assays were performed using a 100ul pipette tip to scratch the cells to make a separate wound. Afterward, wounded cells were washed with PBS, and the remaining cells were cultured in DMEM containing 2% FBS. Migration capacity was evaluated by light microscope by quantifying the area covered by migrated cells at 0 and 48 hours.

### Transwell assays for migration

After the above-mentioned transfection, Transwell migration assays were carried out using a 24-well chamber (Corning). Cells (2 x 10^4^) were suspended in 100ul DMEM and added to the upper layer of chambers. 700ul DMEM containing 10% FBS was added below the chambers. Cells were cultured for 24 hours at 37°C, and then the upper chambers were cleaned with cotton swabs. SW872 cells penetrated and adhered to the bottom of the chamber and were fixed with 4% PFA for 15 min and stained with 0.5% crystal violet for 15 min. Chambers were imaged under a microscope.

### Transwell assays for invasion

After the transfection, Transwell invasion assays were used to examine cell invasion ability. First, 50ul Matrigel (diluted using DMEM containing 10% FBS at 1:8) was loaded in a 24-well chamber (Corning). DMEM containing 10% FBS was added to the lower chamber, and suspension of DMEM containing 5 x 104 cells was added to the upper chamber. After incubation for 24 hours at 37°C, the upper chambers were cleaned with cotton swabs. SW872 cells penetrated and adhered to the bottom of the chamber and were fixed with 4% PFA for 15 min and stained with 0.5% crystal violet for 15 min. Chambers were imaged under a microscope.

### Statistical analysis

The R software (version 4.0.1) and GraphPad Prism (version 9.0.0) were used for statistical analysis. The difference between the two distinct risk groups was compared with the Wilcoxon test. A Chi-square test was used to analyze the clinicopathological characteristics of the two risk groups. The difference in the overall survival rate of STS between the high- and low-risk groups were compared using the Log-rank test. The expression of signature ARGs between normal and STS cell line was evaluated by one-way analysis of variance (ANOVA). The Pearson correlation test was applied to explore the correlation between two variables. A P-value less than 0.05 represent a statistically significant difference.

## Results

### Establishment and validation of the novel ARSig for STS

**Figure 1:**
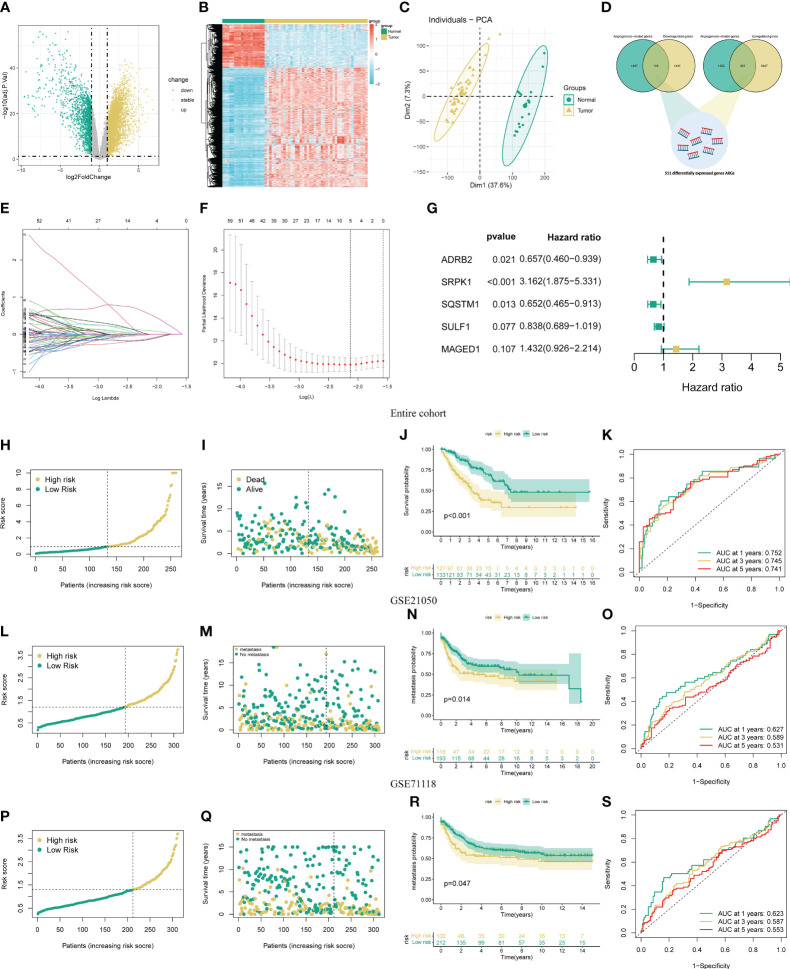
Development and validation of the novel ARSigs for the STS cohort. **(A)** Volcano plot of the DEGs **(B)** Heatmap of the DEGs among tumor and normal tissue. **(C)** Principal component analysis (PCA) based on DEGs to distinguish STS from normal tissues. **(D)** Venn diagram among DEGs and ARGs. **(E)** LASSO regression analysis of 116 prognostic DEARGs **(F)** Cross-validation method to select candidate signature genes. **(G)** Multivariate Cox regression analysis of signature gene. **(H-I)** Risk score curve and survival status distribution of STS cohort in the entire group. **(J)** KM survival analysis of the high and low-risk groups in entire groups. **(K)** Assess the prognostic performance of the novel ARSig using the ROC curve in the entire group. **(L-M)** Risk score curve and survival status distribution in the GSE21050 cohort. **(N)** KM survival analysis of the high and low-risk groups in the GSE21050 cohort. **(O)** Assess the prognostic performance of the novel ARSig using the ROC curve in the GSE21050 cohort. **(P-Q)** Risk score curve and survival status distribution in the GSE71118 cohort. **(R)** KM survival analysis of the high and low-risk groups in the GSE71118 cohort. **(S)** Assess the prognostic performance of the novel ARSig using the ROC curve in the GSE71118 cohort.

The flow chart of our study is presented in **Figure S1**. Initially, we identify 5499 DEGs (3900 upregulated and 1599 downregulated) in the STS cohort through differential expression analysis. The volcano and heat map of these DEGs is presented in **Figures 1A, B**. The PCA analysis indicates that the STS and normal tissue samples could be clearly separated by the combined expression of these DEGs (**Figure 1C**). Next, we obtained 1605 ARGs from previous studies. From the intersection between DEGs and ARGs, we identify 511 DEARGs in STS, including 403 upregulated and 108 downregulated ARGs (**Figure 1D**). The upregulated and downregulated ARGs are shown as cluster heatmaps and volcano plots in **Figure S2**. Subsequently, we find 116 DEARGs relevant to the prognosis of STS by univariate analysis (**Table S8**), which are enrolled for the angiogenesis-related signature construction. For ARSig construction, we first screen the candidate prognostic DEARGs through LASSO Cox regression analysis (**Figures 1E, F**). Next, the multivariate Cox regression analysis is applied to optimize the signature (**Figure 1G**). As a result, the novel ARSig composed of five prognostic DEARGs (ADRB2, SRPK1, SQSTM1, SULF1, and MAGED1) is established. According to the multivariate analysis results (**Table S9**), the formula of ARSig risk score calculation is as follows: Risk score = SRPK1* 1.15110386815651 - ADRB2* 0.420077308273549 - SQSTM1* 0.428083645117686 - SULF1* 0.176496892249047 + MAGED1*0.3588603726472 31. **Figures 1H, I** indicates the risk score and survival status distribution of each STS individual. With the risk score increasing, the number of STS deaths also increases. Consistently, the KM analysis suggests that the STS patients with a lower risk score displayed a significantly improved survival rate than those with a higher risk score (**Figure 1J**). Furthermore, the AUC of the ROC curve for 1-, 3-, and 5-year survival was 0.835, 0.843, and 0.801, respectively, which indicated the predictive power of the novel ARSig (**Figure 1K**).

To estimate the predictive robustness of the novel ARSig, we performed internal validation in the testing and the entire STS cohort. As shown in **Figures S3-4**, we observed similar results in the training and the testing STS cohort. We also use the external cohort (GSE21050 and GSE71118 cohort) to verify the predictive performance of the novel ARSig (**Figures 1L-S**). Consistent with the results from the internal cohort, the distribution plot and Kaplan–Meier survival analysis indicated that the STS in the low-risk group exhibit a better prognosis than those in the high-risk groups. In aggregate, these results confirmed that the novel ARSig had a promising performance in predicting the prognosis of patients with STS.

### Evaluating the performance of novel ARSig

**Figure 2:**
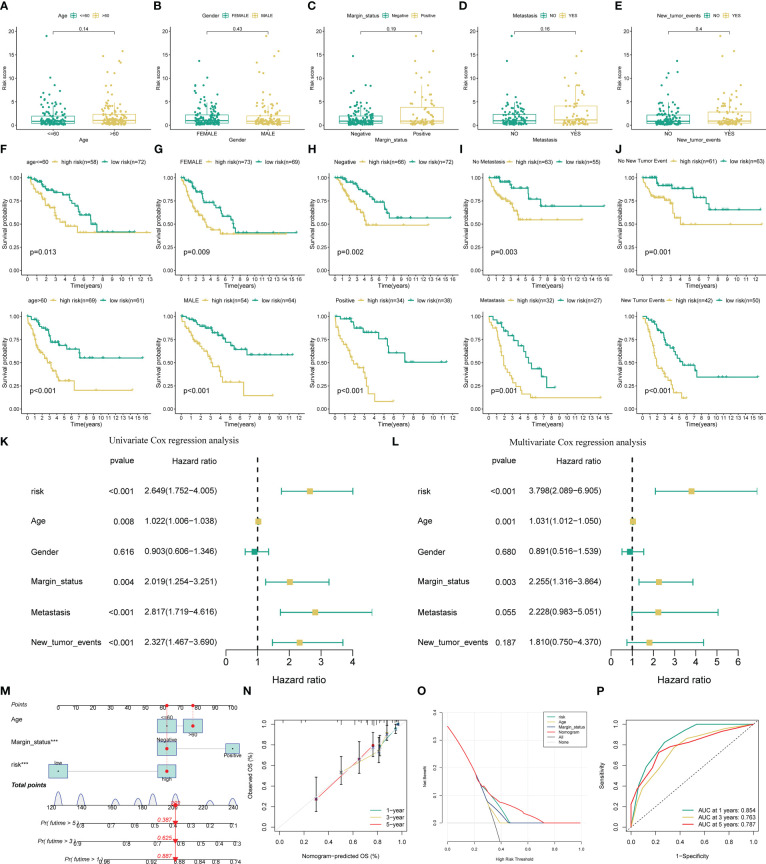
Evaluation of the predictive performance of the novel AGSig. **(A-E)** Boxplots of the risk score in STS were stratified by age, gender, margin status, metastasis, and new tumor events, respectively. **(F-J)** Prognostic value of risk score in patients with different ages, gender, margin status, metastasis, and new tumor events, respectively. **(K)** Univariate Cox regression analysis of angiogenesis-related risk score and clinical characteristics. **(L)** Multivariate Cox regression analysis of angiogenesis-related risk score and clinical characteristics. **(M)** A nomogram based on ARSig risk score and independent clinical factor for predicting 1-, 3-, and 5-year OS of STS. **(N)** Calibration curves. **(O)** The ROC curves for nomogram. **(P)** Decision curve analysis plot.

To determine the prognostic generality of the novel ARSig, we further compared the risk score between distinct clinical subgroups and carried out a subgroup KM survival analysis. There was no significant difference in the risk score distribution between the distinct clinical subgroup, indicating that the novel ARSig was relatively independent of the clinical characteristics (**Figures 2A-E**, **S5**). In addition, the subgroup survival analysis demonstrates that the low-risk group patients have an improved OS comparing to the high-risk subgroup in distinct clinical features (age, gender, margin status, metastasis status, and new tumor events. **Figures 2F-J**). Importantly, we also implement univariate and multivariate Cox regression analyses to investigate whether the novel ARSig is an independent prognostic factor for STS patients. The univariate analysis indicates that the risk score, age, margin status, metastasis, and new tumor events are remarkably associated with OS (**Figure 2K**). Encouragingly, the multivariate analysis result further confirmed that the ARSig risk score is an independent prognostic indicator affecting the OS of STS (**Figure 2L**). Moreover, we also found that the c-index of our signatures based on ARGs performs better than almost all previous signatures (**Figure S6**).

To facilitate the clinical application of the novel ARSig, we further construct a nomogram incorporating the ARSig risk score and independent clinical factor. According to the nomogram, we could precisely estimate the 1-year, 3-year, and 5-year survival rates of each STS individual (**Figure 2M**). Encouragingly, the calibration curves exhibits that the actual values of the 1-, 3-, and 5-year OS match those predicted by the nomograph, indicating the nomogram we built is reliable and accurate (**Figure 2N**). The 1-, 3-, and 5-year area under the ROC curve of the nomogram are 0.854, 0.763, and 0.787, respectively (**Figure 2O**). Also, the DCA demonstrates that the nomogram has the best clinical net benefit comparing with other variables (**Figure 2P**). Overall, these findings show that the novel ARSig is successfully constructed and exhibited reliable and has excellent performance for the OS prediction of STS.

### The signature ARGs in STS

**Figure 3:**
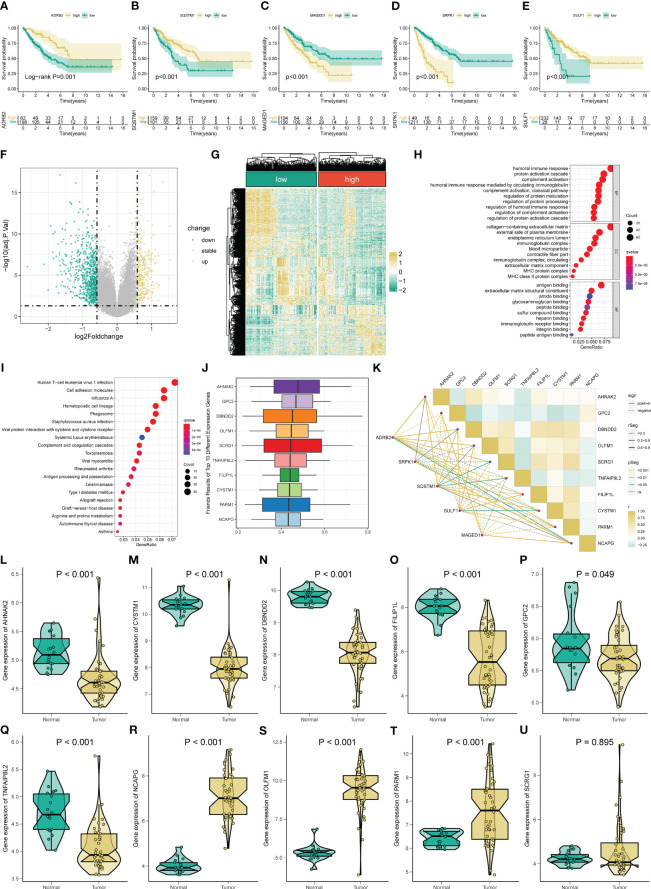
Gene functional enrichment analysis of differentially expressed genes between distinct risk groups. KM survival curves for ADRB2 **(A)**, SQSTM1 **(B)**, MAGED1 **(C)**, SRPK1 **(D)**, and SULF1 **(E)**. **(F-G)** The volcano plot and heatmap of DEGs among the low- and high-risk risk group. **(H)** GO enrichment analysis includes a biological process (BP), cellular component (CC), and molecular function (MF). **(I)** KEGG enrichment analysis indicates related genes and pathways. **(J)** The Friends analysis of GO-related genes. **(K)** The correlation between these ten hub genes and each signature ARG. **(L-U)** The expression of these ten hub genes in STS.

Subsequently, we perform the KM survival analysis to investigate the respective prognostic value of each signature ARG. Similarly, we find that the STS patient with mitigation of ADRB2 and SQSTM1 has poorer OS (**Figures 3A, B**), while the augmented levels of MAGED1, SRPK1, and SULF1 seem to account for a better prognosis in STS (**Figures 3C-E**). Collectively, these results imply that the abnormal expression of these signature ARGs might be relevant to the prognosis of STS.

### Functional enrichment analysis and angiogenesis-related hub genes in STS

To comprehend the difference in the functional pathways among the distinct risk groups, we identify 1006 DEGs between the low- and high-risk groups (**Figures 3F, G**). Then, the functional enrichment analysis is conducted based on these DEGs. The GO analysis results indicate that these DEGs are mainly enriched in immune-related functions, like humoral immune response, humoral immune response mediated by circulating immunoglobulin, regulation of humoral immune response, immunoglobulin complex, and immunoglobulin receptor binding (**Figure 3H**). Also, **Figure 3I** shows the top twenty pathways these DEGs enriched. Among them, the Human T−cell leukemia virus 1 infection, Viral protein interaction with cytokine and cytokine receptors, and Antigen processing and presentation are immune-related, while the Cell adhesion molecules are associated with tumorigenesis. Moreover, we define ten potential hub genes (AHNAK2, GPC2, DBNDD2, OLFM1, SCRG1, TNFAIP8L2, FILIP1L, CYSTM1, PARM1, and NCAPG) in the identified angiogenesis-associated GO progress through the Friend analysis (**Figure 3J**). We observe a remarkably co-expression relevance between the signature ARGs and these ten hub genes (**Figure 3K**). Almost all these hub genes display an abnormal expression in the STS compared to normal tissue, except for SCRG1 (**Figures 3L-U**). Equally, the KM survival also suggests that all ten hub genes exhibit significant prognostic effects in STS (**Figure S7**).

### Exploring the underlying pathways in STS

**Figure 4:**
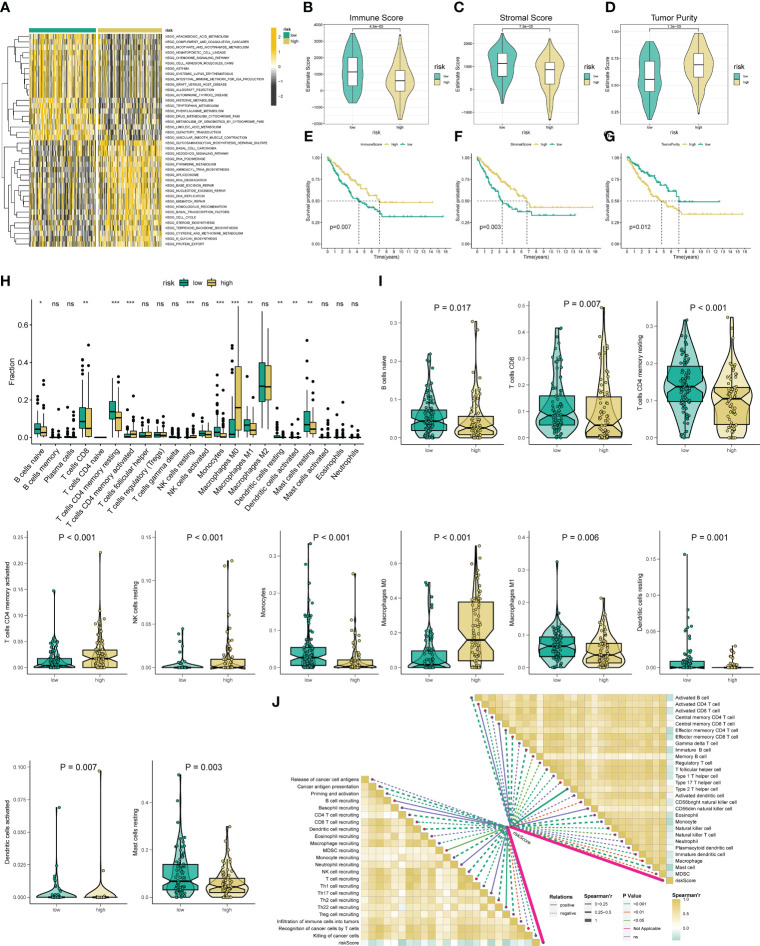
Analysis of immune status based on the angiogenesis-related risk score. **(A)** Heat maps of GSVA exhibit signaling pathways between low- and high-risk groups. **(B-D)** Comparison of immune, stromal, and tumor purity scores between the high- and low-risk groups. **(E-G)** Prognostic value of immune, stromal, and tumor purity score in STS. **(H)** The abundance of 22 infiltrating immune cell types in the two risk subgroups. **(I)** The proportion of B cells naive, T cells CD8, T cells CD4 memory resting, T cells CD4 activated, NK cell resting, Monocytes, Macrophage M0, Macrophage M1, Dendritic cell resting, Dendritic cell activated, and Mast cell resting in the different risk groups. **(J)** The correlation between the ARSig risk score and the infiltration of immune cells. * represent P < 0.05, ** represents P < 0.01, *** represents P < 0.001, and ns represent no significance.

To further verify the molecular mechanism difference between the distinct risk groups, we perform the GSEA and GSVA analysis. The GSEA shows that the high-risk STS patient mainly associated with tumorigenesis pathways, such as basal cell carcinoma, cell cycle, DNA replication, hedgehog signaling pathway, and Wnt signaling pathway (**Figure S8A**). Meanwhile, those mainly enriched pathways in the low-risk group are relevant to immunity function (**Figure S8B**). In the following GSVA analysis, we obtain results consistent with the previous GSEA, such as the low risk mainly concentrated in complement and coagulation cascades, chemokine signaling pathway, and graft versus host disease (**Figure 4A**). Altogether, these results provide promising clues for inferring the underlying mechanism of the novel ARSig regulating STS progress.

### TME and immune cell infiltration analysis

Given these above functional enrichment analysis results and the critical role of tumor immunity in tumor development, we further investigate the immune status among the various ARSig risk groups. Initially, the ESTIMATE analysis indicates that the low-risk STS patients displayed an enhanced immune and stromal score and a lower tumor purity score, hinting the STS cohort in the low-risk group has a better immune infiltration (**Figures 4B-D**). Also, we find that both the patients with an augmented immune and stromal score or an attenuated tumor purity score exhibits an ameliorated prognosis (**Figures 4E-G**). Subsequently, we evaluate the infiltrate proportion of the 22 types of immune cells in STS by applying the CIBERSORT algorithm (**Figure S9A**). We observe that the abundance of naive B cells, CD8 T cells, CD4 memory resting T cells, Monocytes, M1 Macrophage, resting dendritic cells, and resting mast cells are elevated in the low-risk groups, while the infiltration level of CD4^+^ T cells, Resting NK cells, M0 Macrophage, and activated dendritic cell in the low-risk group is lower than those in the high-risk groups (**Figures 4H, I**). Besides, there are remarkable correlations between the ARSig risk score and signature ARGs with the proportion of the immune cell infiltration (**Figures 4J**; **S9B**). Notably, the KM survival demonstrates an enhanced infiltration level of naive B cells, activated NK cells and CD8 T cells are relevant to an improved prognosis in STS (**Figures S9C-E**). Contrary, the patients with an increasing abundance of M0 Macrophage, M2 Macrophage, and CD4+ T cells have a poorer OS (**Figures S9F-H**).

**Figure 5:**
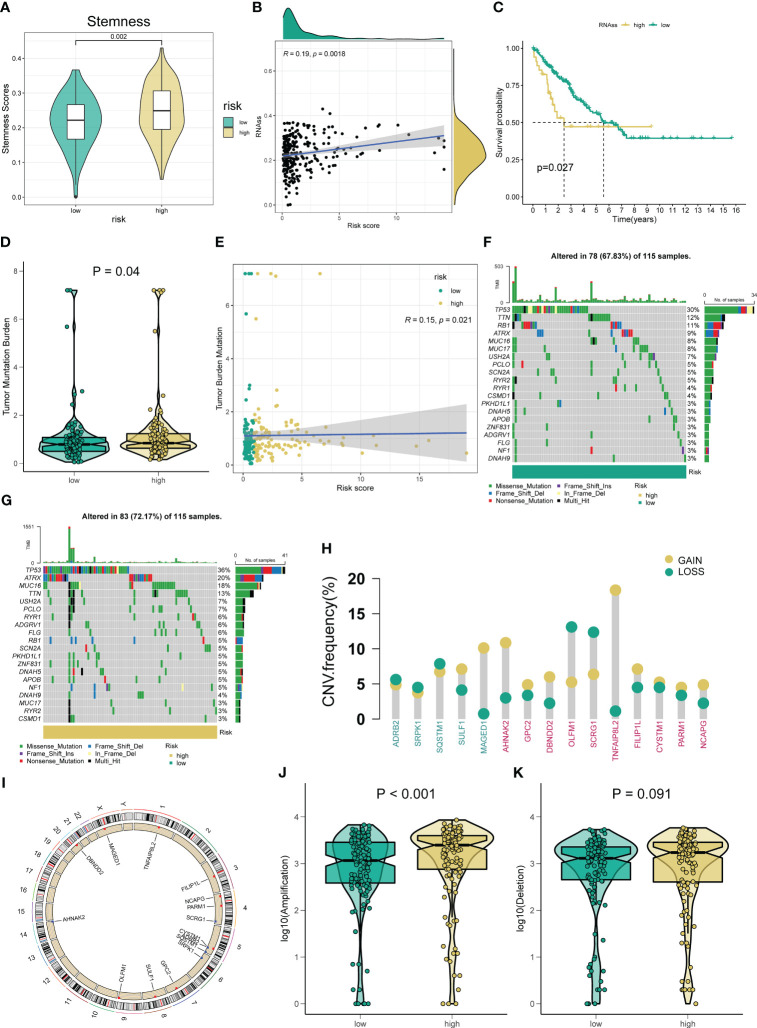
Correlation between the novel ARSig and Tumor mutation status. **(A-C)** The relationships between angiogenesis-related risk score and CSC index. **(D)** TMB score among different risk groups. **(E)** The Spearman correlation analysis of the angiogenesis-related risk score and TMB score. **(F-G)** The difference in Mutations between distinct risk groups (the top 20 mutated genes). **(H)** Frequencies of CNV gain, loss, and non-CNV among signature ARGs and ten hub genes. **(I)** The location of signature ARGs and ten hub genes on chromosomes. **(J-K)** The difference in CNV loss and gain between the low- and high-risk groups.

### Association of the novel ARSig with tumor mutation burden

Considering the importance of CSC and TMB in tumor generation and development, we explore their association with the novel ARSig. **Figures 5A–C** indicates the relationship between ARSig risk scores and the CSC index. We find that risk score is positively correlated with the CSC index, and the STS patients with a lower CSC index exhibits an ameliorated prognosis. For TMB, the higher risk is correlated to an elevated TMB score (**Figures 5D, E**). Also, the waterfall plot indicates that TP53, TTN, and RB1 are the top three mutation rate genes in the low-risk group (**Figure 5F**). Similarly, TP53 shows the highest mutation frequency in the high-risk group, followed by ATRX and MUC16 (**Figure 5G**). Then, we investigate somatic copy number alterations in these signature ARGs and hub genes. Among them, MAGED1, AHNAK2, and TNFAIP8L2 have widespread CNV increases, while OLFM1 and SCRG1 display CNV decreases (**Figure 5H**). The locations of the CNV alterations in these genes on their respective chromosomes are presented in **Figure 5I**. We further observe that the high-risk group company with an elevated frequency of copy number amplification compared to the low-risk group (**Figures 5J, K**).

### Prediction efficacy of the immunotherapy and chemotherapy

**Figure 6:**
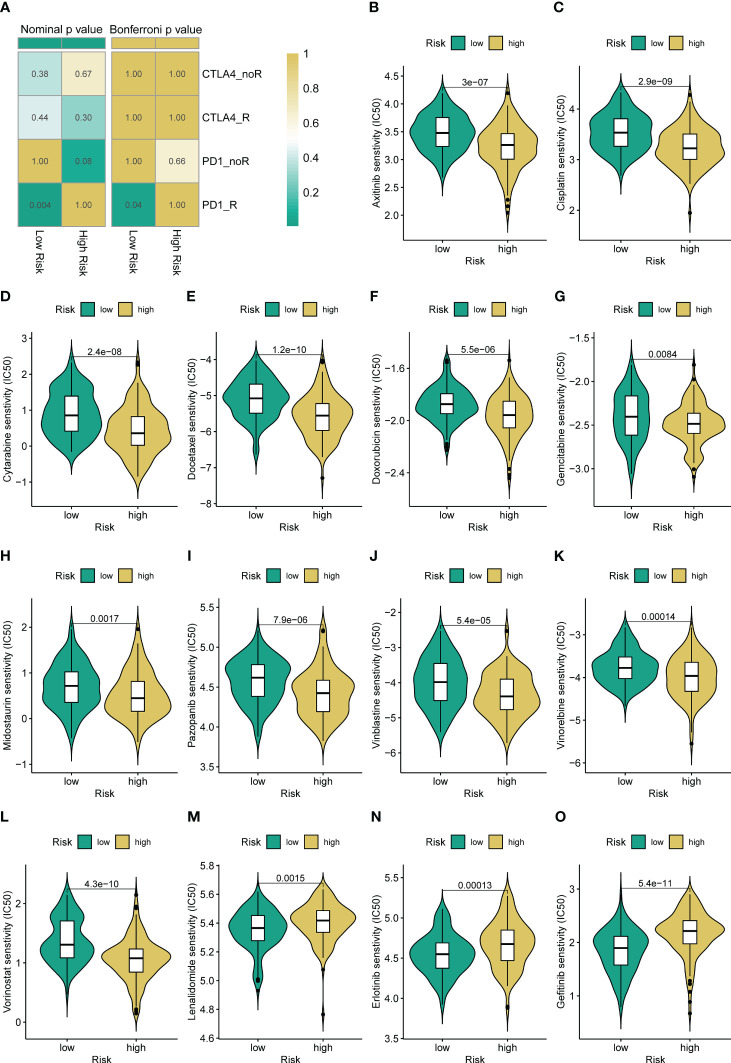
Different immunotherapy and chemotherapy sensitivity analyses. **(A)** The immunotherapy responses to immune checkpoint inhibitors in the STS cohort with a different risk score. **(B-O)** Relationships between ARSig risk score and chemotherapeutic sensitivity.

Immune checkpoint modulators are known to play a critical role in tumor immunity and immunotherapy. We find that the expression of virtually all immune checkpoints is upregulated in the low-risk group compared with the high-risk group (**Figure S10**). Therefore, we further assess the response to immune checkpoint inhibitors (CTLA4-blocker and PD1-blocker) in the subgroup classified by ARSig risk score. As present in **Figure 6A**, the STS patients in the low-risk groups have a better response to PD1-blocker (Bonferroni P-value < 0.05). Equally, we estimate the response of the STS cohort to commonly used chemotherapeutic agents by comparing the difference in IC50 between the distinct risk groups. The STS cohort in the low-risk group has a higher IC50 of axitinib, cisplatin, cytarabine, docetaxel, doxorubicin, gemcitabine, midostaurin, pazopanib, vinblastine, vinorelbine, and vorinostat than those in the high-risk group (**Figures 6B-L**). In contrast, the IC50 of lenalidomide, erlotinib, and gefitinib in the low-risk group is lower than those in the high-risk group (**Figures 6M-O**).

### The effect of signature ARGs in STS

**Figure 7:**
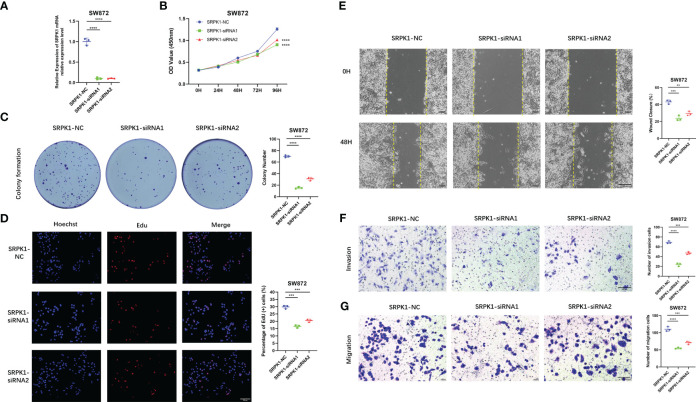
Down-regulated SRPK1 inhibits soft tissue sarcoma proliferation, migration, and invasion. **(A)** SRPK1 was transfected with siRNA for 48 hours. **(B)** The cell proliferation rate of NC, SRPK1-siRNA1, and SRPK1-siRNA2 groups were detected by CCK-8 assay. **(C)** Colony formation abilities in NC, SRPK1-siRNA1, and SRPK1-siRNA2 groups. Colony numbers were shown in the corresponding column at the right. **(D)** The cell proliferation rate of NC, SRPK1-siRNA1, and SRPK1-siRNA2 groups was detected using Edu-assay. Percentages of Edu-positive cells were quantified in corresponding columns at right. **(E, F)** The migration ability of NC, S SRPK1-siRNA1, and SRPK1-siRNA2 groups was illustrated by scratch tests and transwell assay for migration. **(G)** The invasion abilities of NC, SRPK1-siRNA1, and SRPK1-siRNA2 groups were demonstrated using transwell assay for invasion. ** represents P < 0.01, *** represents P < 0.001, and **** represents P < 0.0001.

**Figure 8:**
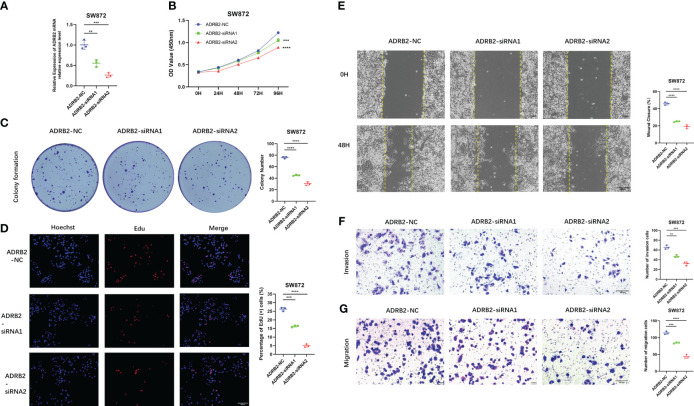
Down-regulated ADRB2 inhibits soft tissue sarcoma proliferation, migration, and invasion. **(A)** ADRB2 was transfected with siRNA for 48 hours. **(B)** The cell proliferation rate of NC, ADRB2-siRNA1, and ADRB2-siRNA2 groups was detected by CCK-8 assay. **(C)** Colony formation abilities in NC, ADRB2-siRNA1, and ADRB2-siRNA2 groups. Colony numbers were shown in the corresponding column at the right. **(D)** The cell proliferation rate of NC, ADRB2-siRNA1, and ADRB2-siRNA2 groups was detected using Edu-assay. Percentages of Edu-positive cells were quantified in corresponding columns at right. **(E, F)** The migration ability of NC, ADRB2-siRNA1, and ADRB2-siRNA2 groups was illustrated by scratch tests and transwell assay for migration. **(G)** The invasion abilities of NC, ADRB2-siRNA1, and ADRB2-siRNA2 groups were demonstrated using transwell assay for invasion. ** represents P < 0.01, *** represents P < 0.001, and **** represents P < 0.0001.

Importantly, we verify the expression of each signature ARG in the STS cell lines using RT-qPCR. As shown in **Figure S11**, we observe that the whole signature ARGs are significantly dysregulated in STS cell lines. Considering that ARDB2 and SRPK1 are aberrantly elevated in the STS, we further explore the function of ARDB2 and SRPK1 in STS. As shown in **Figures 7A**, **8A**, the expressions of SRPK1 and ARDB2 were significantly down-regulated in SW872 cells after siRNA transfection. The CCK8 results show that the attenuation of SRPK1 and ARDB2 could lead to the slowing down of the proliferation rate of SW872 (**Figures 7B**, **8B**). Consistently, the colony-forming ability of SW872 is attenuated with the downregulation of SRPK1 and ARDB2 (**Figures 7C**, **8C**). Also, compared to negative control groups, the percentage of EdU-positive cells exhibits a downward trend in the siRNA-SRPK1 and siRNA-ARDB2 groups (**Figures 7D**, **8D**). On the other hand, the scratch test indicates that the moving distance of SW872 in the siRNA-SRPK1 and siRNA-ARDB2 group was significantly less than that of the control group (**Figures 7E**, **8E**). Moreover, the transwell migration and invasion assay reveal that the SRPK1 and ARDB2 diminished could inhibit SW872 cell migration and invasion (**Figures 7F, G**, **8F, G**). Hence, these above-mentioned results imply that the abnormal overexpression of ARDB2 and SRPK1 could promotes the malignant phenotype of soft tissue sarcoma cells, further validating our bioinformatic analysis results.

## Discussion

STS is a heterogeneous malignant disease deriving from mesenchymal, constituting 1% of adult malignancies and 15% of malignant neoplasms in childhood (26). Since the aggressiveness, metastasis, and relapse of tumor, the overall survival rates of STS remain suboptimal. Therefore, it is critical to establish an effective prognostic biomarker for risk stratification and precision prognostic prediction of STS. Angiogenesis has been revealed to play a crucial role in carcinogenesis and progression, which is highly dependent on angiogenic cytokines (27, 28). For instance, the secretion of VEGF is essential to tumor vascularization, and its inhibition disrupts tumor progression (29). HIF1 is a heterodimeric protein consisting of HIF1α and HIF1β subunits, and it is also known to be an important stimulus for tumor angiogenesis (30). In addition, several recent research has demonstrated that the angiogenesis-related gene signature was closely linked to the prognosis of various cancer patients. Xin Qing et al. identified an angiogenesis-associated genes signature, contributing to predicting the prognosis, clinical characteristics and TME of gastric cancer (12). Similarly, the angiogenesis-related gene signature exhibited a promising ability for the prognosis and treatment response prediction of glioblastoma multiforme and will help the therapeutic strategies selection in glioblastoma multiforme (11). However, numerous studies have only evaluated the role of single ARGs in STS. The research systematically elucidates the holistic impact of the combinatorial of diverse ARGs is still lacking.

In the present study, we identified 116 DEARGs with prominent prognosis significance of STS. Subsequently, a novel ARSig consisting of five angiogenesis-associated genes was successfully established using LASSO, univariate, and multivariate COX regression analysis. The novel prognostic ARSig exhibited an effective ability to stratify the prognosis of STS. Our results show that the STS patients in the low-risk groups have an improved prognosis, while the prognosis of STS in the high-risk group is significantly poorer. Next, the prediction performance of the novel ARSig is further confirmed using the ROC curve, internal validation, and subgroup survival analysis. In addition, the univariate and multivariate Cox analysis demonstrate that the ARSig risk score is an independent prognostic predictor for the OS of STS. Encouragingly, a consistent validation result in predicting OS is also founded in the external cohort (GSE21050 and GSE71118), which further corroborate the reliability and potential of our signature. Herein, we construct a novel prognostic signature based on ARGs, which could be used as a reliable and independent marker to help conduct personalized prognostic evaluations in STS.

To further investigate the association of the novel ARSig with STS, we explore the difference in underlying mechanisms between the two distinct risk groups using GSEA and GSVA. Interestingly, we observe that the GSEA and GSVA results both show that the STS patients with a higher risk score mainly enriched in cell cycle, DNA replication, and hedgehog signaling pathway. As is known to all, growing evidence has confirmed that these pathways are involved in the progression of various tumors. For instance, PLA2G10 could promote the cell cycle progression of soft tissue leiomyosarcoma cells through upregulated of the expression of cyclin E1 and CDK2 (31). The dysregulated of DNA replication results in abnormal gene phenotypes that trigger normal cells to transform into malignant ones (32). In addition, the hedgehog signaling pathway also plays a vitally important role in the tumor. Dongdong Cheng et al. prove that CNOT1 cooperates with LMNA to aggravate the occurrence of osteosarcoma by regulating the Hedgehog signaling pathway (33). On the contrary, the patients in the low-risk group seem relevant to immune-related responses, which may affect the tumor immunity microenvironment of STS. Given these results and previous studies, it is reasonable to believe that these identify pathways provided novel insights into the relationship between the novel ARSig and tumor biology of STS.

Meanwhile, ten key hub genes (AHNAK2, GPC2, DBNDD2, OLFM1, SCRG1, TNFAIP8L2, FILIP1L, CYSTM1, PARM1, and NCAPG) are determined using the Friend analysis, which is associated with the prognosis of STS. The Friends analysis is a commonly used method for identifying hub genes in the pathway (34). Surprisingly, the functional role of these ten hub genes in tumor has been widely reported in previous studies. AHNAK2 has been shown to be a prognostic marker in papillary thyroid cancer, clear cell renal cell carcinoma (ccRCC), and lung adenocarcinoma (35–37). Minglei Wang et al. reveal that the overexpression of AHNAK2 could drive tumorigenesis and progression of ccRCC by facilitate EMT and cancer cell stemness (36). FILIP1L is a tumor suppressor with diminished expression in various tumors (38). For instance, the downregulation of FILIP1L causes the aberrant stabilization of a centrosome-associated chaperone protein, thereby driving aneuploidy and progression in colorectal adenocarcinoma (39). Guoming Chen et al. demonstrate that GPC2 could sreve as a Potential prognostic, diagnostic, and immunological biomarker in pan-cancer (40). In addition, it is revealed that the elevated TNFAIP8L2 inhibit the survival and proliferation of colorectal cancer cell line, while endogenous TNFAIP8L2 facilitate the tumorigenesis when exposure to dangerous environment (41). NCAPG is overexpressed in cardia adenocarcinoma (CA), which could suppress the apoptosis and advocate the epithelial-mesenchymal transition of the CA cell line via activating the Wnt/β-catenin signaling pathway (42). Consistently, OLFM1 could inhibit the growth and metastasis of colorectal cancer cells through affect the NF-κB signalling pathway (43). Also, the oncogenic potential and important role of PARM1 in leukemogenesis were proved by Cyndia Charfi et al., which could promote anchorage and cell proliferation capacity (44). However, research on the role of SCRG1, DBNDD2 and CYSTM1 in tumorigenesis and development is currently lacking. Collectively, these hub genes exhibit a significant association with tumors, representing a promising clue for future biomarker research in STS.

It has shown that the tumor immune microenvironment is closely relevant to the progression and invasion, with the tumor immune microenvironment receiving considerable attention past few years (45). In the low-risk group, the immune and stromal scores and the abundance of immune infiltration augmented significantly, indicating the STS cohort with a low-risk score has a better immune status. Consistently, previous research has demonstrated that immune infiltration is an ignored prognostic factor for tumor (46), and the ameliorated immunity status was related to the prognosis of STS (47). Interestingly, we observe a decreased M0 infiltration and enhanced M1 macrophage infiltration degree in the low-risk group, and the STS patient with more M0 and M2 macrophage infiltration degrees has an attenuated prognosis. As we all know, macrophages are very versatile cells with a high degree of plasticity and have various functions in various pathological processes (48). Macrophages are broadly categorized into M1 classically activated macrophages, and M2 alternatively activated macrophages (49). Among them, M1 macrophages have anti-tumour effects, while M2 macrophages have pro-tumour effects (50). Therefore, it is reasonable to believe that the infiltration degree of macrophages may partly account for the different tumor immune microenvironment among distinct risk groups, and the different immune status is closely correlated with the prognosis of STS in different ARGsig risk groups.

Recently, immunotherapy has become a promising strategy, which is expected to become the predominant anti-tumor treatment in the future (51). However, not all malignancies benefit from immunotherapy (52). Therefore, stratifying and differentiating patients is necessary for the effectiveness of immunotherapy (53). In the present study, we observe that the low-risk STS patients had an elevated expression of immune checkpoint genes. Similarly, the STS cohorts with low ARSig risk scores exhibits a positive response for anti-PD1, indicating the novel ARSig has a potential ability to predict response to immunotherapy in STS. Also, chemotherapy is another important alternative therapeutic method for patients with STS (54). We find that the low-risk STS cohort responded better to lenalidomide, erlotinib, and gefitinib, while the high-risk STS patients are more sensitive to axitinib, cisplatin, cytarabine, docetaxel, doxorubicin, gemcitabine, midostaurin, pazopanib, vinblastine, vinorelbine, and vorinostat. It may help clinicians choose an appropriate chemotherapy plan based on the risk score. In general, the novel ARSig we presented may provide insight into the individualized immunotherapy and chemotherapy of STS.

Notably, we finally detect the expression levels and the effect of signature ARGs using *in vitro* experiment in the STS cell line, and the result shows that there was a significant difference in the expression of these ARGs among the STS and control cells, increasing the credibility of our study. It is worth mentioning that some ARGs have been demonstrated to be associated with the malignant progression of cancer. For example, the ARDB2 signaling could facilitate the progression and sorafenib resistance of hepatocellular carcinoma via inhibited autophagic degradation of HIF1α (55). SRPK1 is frequently overexpressed in gastric cancer, resulting in tumor cell growth by regulating the small nucleolar RNA expression (56). Consistently, our study reveals that ARDB2 and SRPK1 could promote the proliferation, migration, and invasion ability of SW872. As a member of ARGs, the specific mechanism by which SRPK1 and ARDB2 play a role in angiogenesis is also worth exploring. Currently, studies have reported that the inhibition of SRPK1 can reduce the expression of pro-angiogenic VEGF, thereby maintaining the production of anti-angiogenic VEGF isoforms (57). Also, Yingwei Chang et al. proved that the SRPK1 could affect the angiogenesis via the PI3K/Akt signaling pathway (58). However, the mechanism of ARDB2 in angiogenesis remains unclear. Hence, these results further confirm the reliability of our study, but the specific mechanisms of ARDB2 and SRPK1 in the angiogenesis of STS are worth further exploration in the future.

## Conclusion

Briefly, our study reveals that the identified ARSig is a robust prognostic marker for OS prediction in patients with STS. Furthermore, the stratification base on the novel ARSig could guide the clinical decision, tumor immune microenvironment prediction, personalized immunotherapy and chemotherapy of STS. It is reasonable to believe that our study offers a valuable basis for further research.

## Data availability statement

The datasets presented in this study can be found in online repositories. The names of the repository/repositories and accession number(s) can be found within the article/**Supplementary Materials**.

## Author contributions

SH and ZL contributed to the conception and made final approval of the version, BL performed the study concept and design and wrote the manuscript. CL performed the experiment. CF, HW, HZ, and CT helped with data analysis. All authors contributed to the manuscript revision, read, and approved the submitted version.

## Glossary

**Table:**

STS	Soft tissue sarcoma
VEGF	Vascular endothelial growth factor
HIFs	Hypoxia-inducible factors
FGFs	Fibroblast growth factors
ARGs	Angiogenesis-related genes
ARSig	Angiogenesis-related signatures
CNV	Copy number variation;
TCGA	The Cancer Genome Atlas
GEO	Gene Expression Omnibus
DEARGs	Differential Expressed ARGs
DEGs	Differentially expressed gene
PCA	Principal component analysis
LASSO	least absolute shrinkage and selection operator
OS	Overall survival
KM	Kaplan–Meier
ROC	Receiver operating characteristic
AUC	Area under the curve
GO	Gene Ontology
KEGG	Kyoto Encyclopedia of Genes and Genomes
GSEA	Gene Set Enrichment Analysis
GSVA	Gene set variation analysis
TME	tumor microenvironment
ESTIMATE	Estimation of STromal and Immune cells in Malignant Tumor tissues using Expression data
TMB	Tumor mutation burden
CSC	Cancer Stem Cell
SubMap	Subclass Mapping
IC50	Half of the maximum inhibitory concentration
DMEM	Dulbecco’s modified Eagle’s medium
RT-qPCR	Quantitative reverse transcription PCR
EdU	5-Ethynyl-2’-Deoxyuridine
