# Supplementary material for: Integrative profiling analysis reveals prognostic significance, molecular characteristics, and tumor immunity of angiogenesis-related genes in soft tissue sarcoma
Source: Front Immunol. 2023 Jun 12;14:1178436. doi: 10.3389/fimmu.2023.1178436 (PMC10291125; doi:10.3389/fimmu.2023.1178436)
Supplement: Supplementary file 1 [file DataSheet_1.docx]

**Supplementary Figures**

**
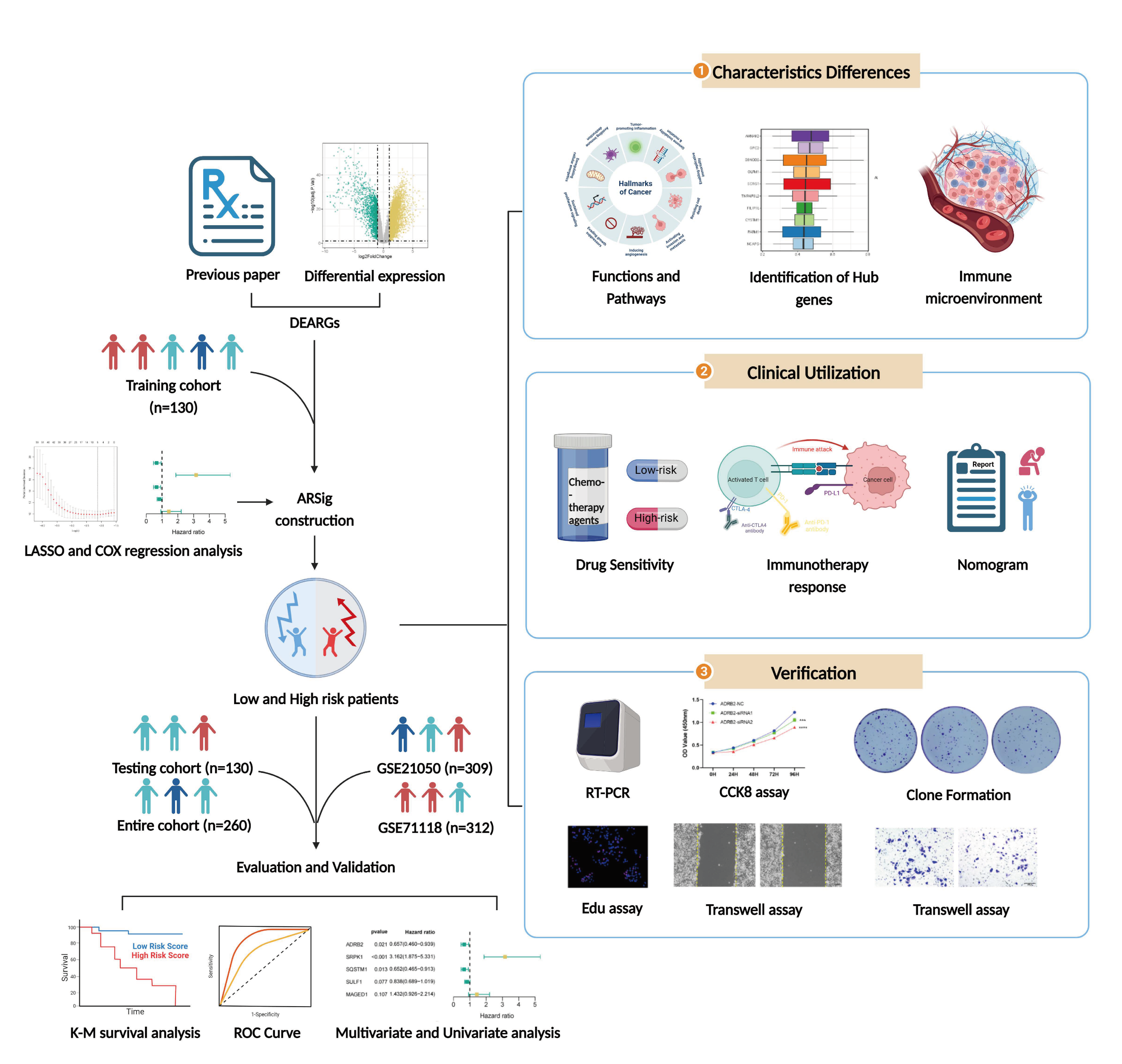
**

**Figure S1.** The flow diagram of our study.


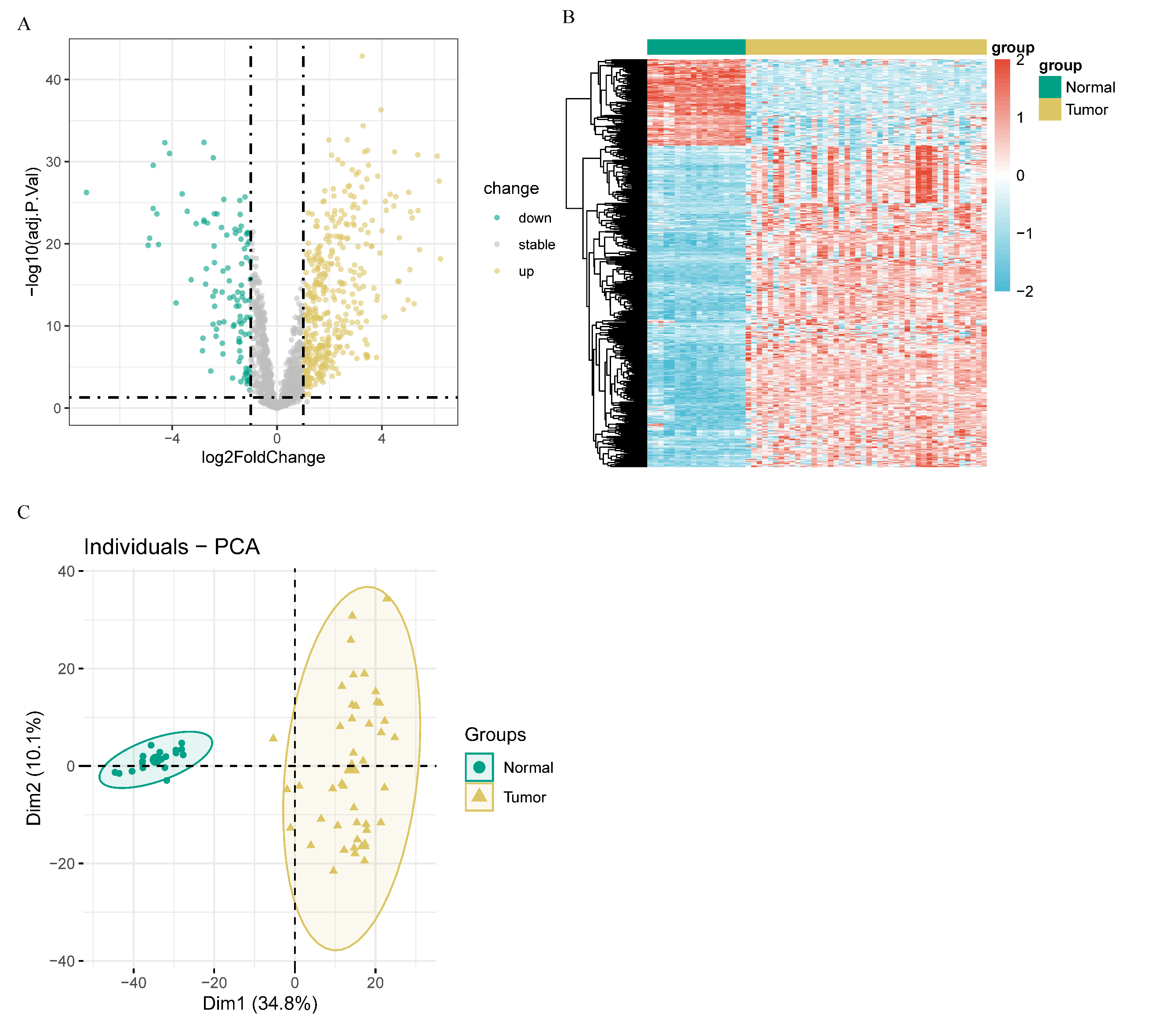


**Figure S2.** DEARGs in STS. (A) Volcano plot of the DEARGs. (B) Heatmap of the DEARGs among tumor and normal tissue. (C) Principal component analysis (PCA) based on DEARGs to distinguish STS from normal tissues.

**
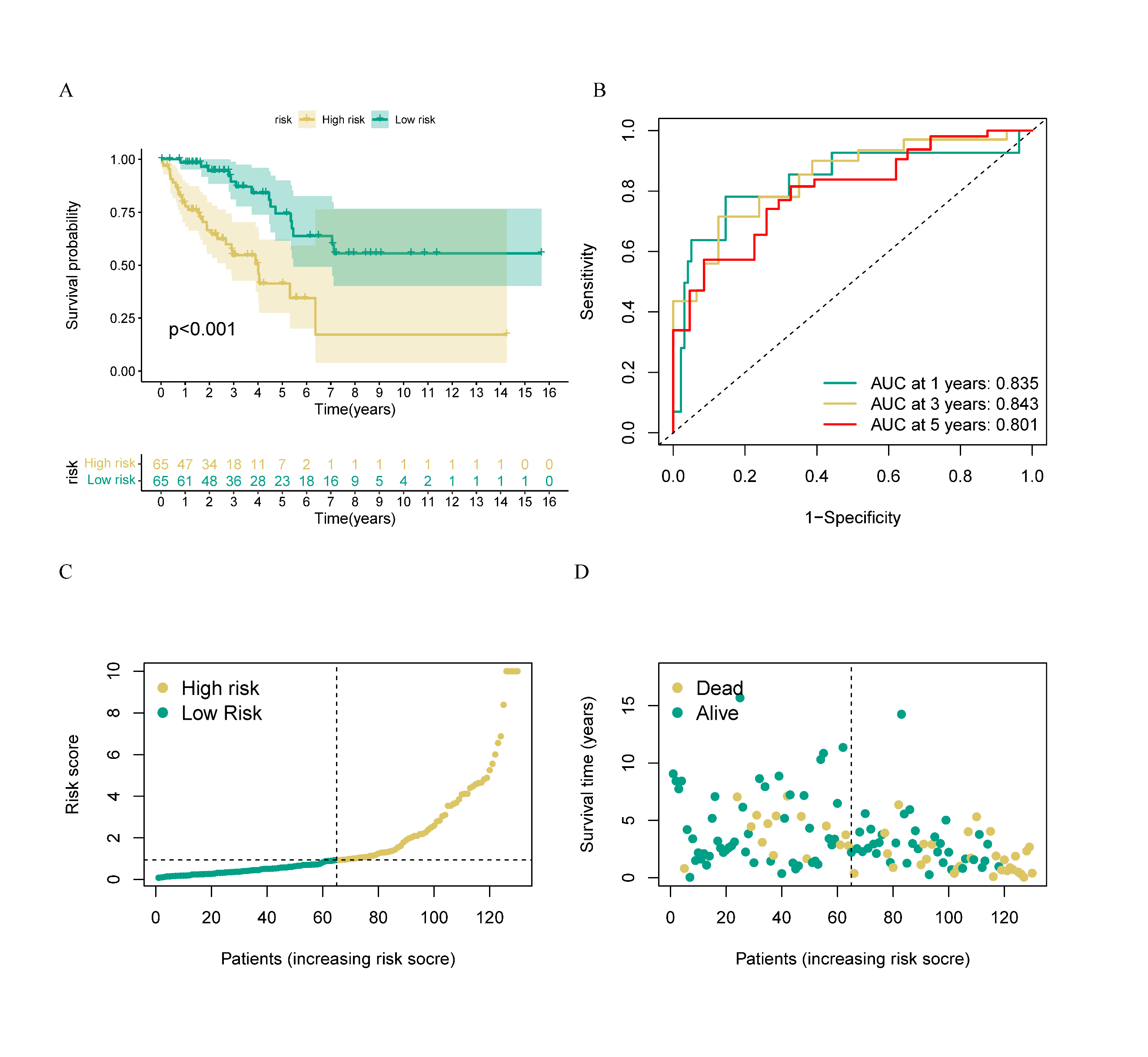
**

**Figure S3.** Validation of the novel ARSig in training cohort. (A) The K-M analysis between the distinct risk groups in the training cohort. (B) ROC curves verified the prognostic performance of the novel signature in the training cohort. (C-D) The distribution of the risk scores and the distributions of overall survival status and risk score in the training cohort.

**
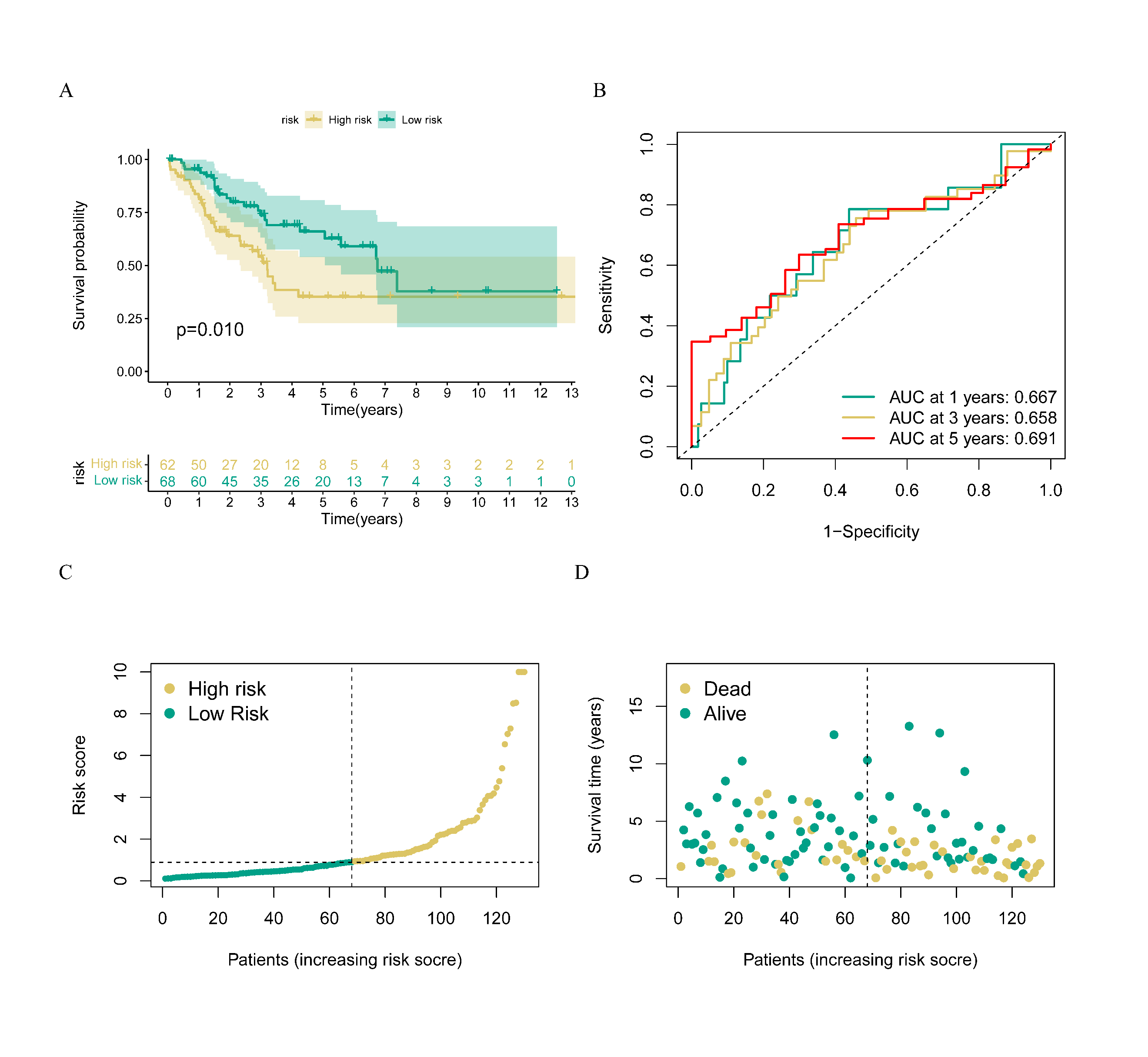
**

**Figure S4.** Validation of the novel ARSig in testing cohort. (A) The K-M analysis between the distinct risk groups in in testing cohort. (B) ROC curves verified the prognostic performance of the novel signature in testing cohort. (C-D) The distribution of the risk scores and the distributions of overall survival status and risk score in testing cohort.


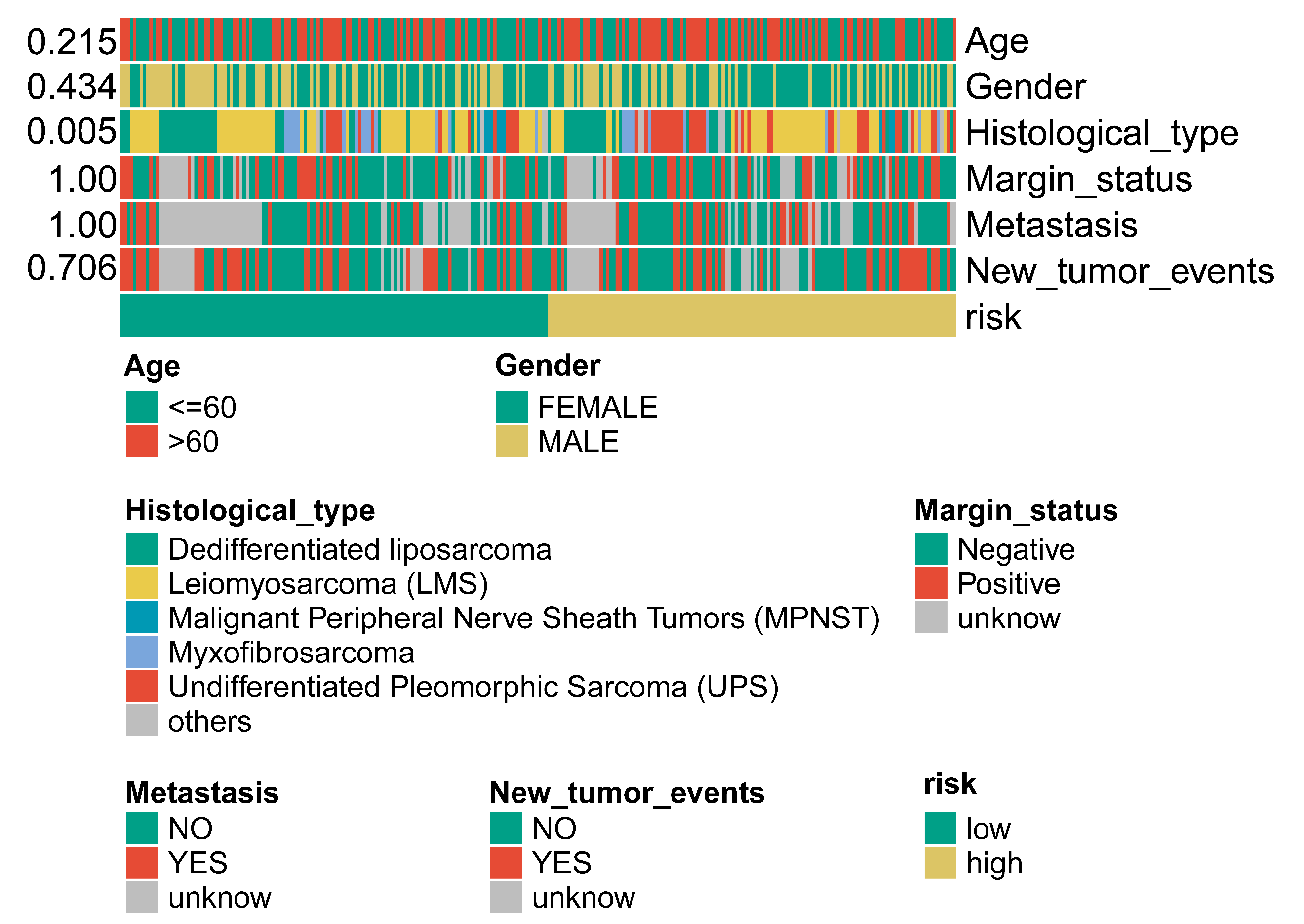


**Figure S5.** Association between the risk scores and clinical features.


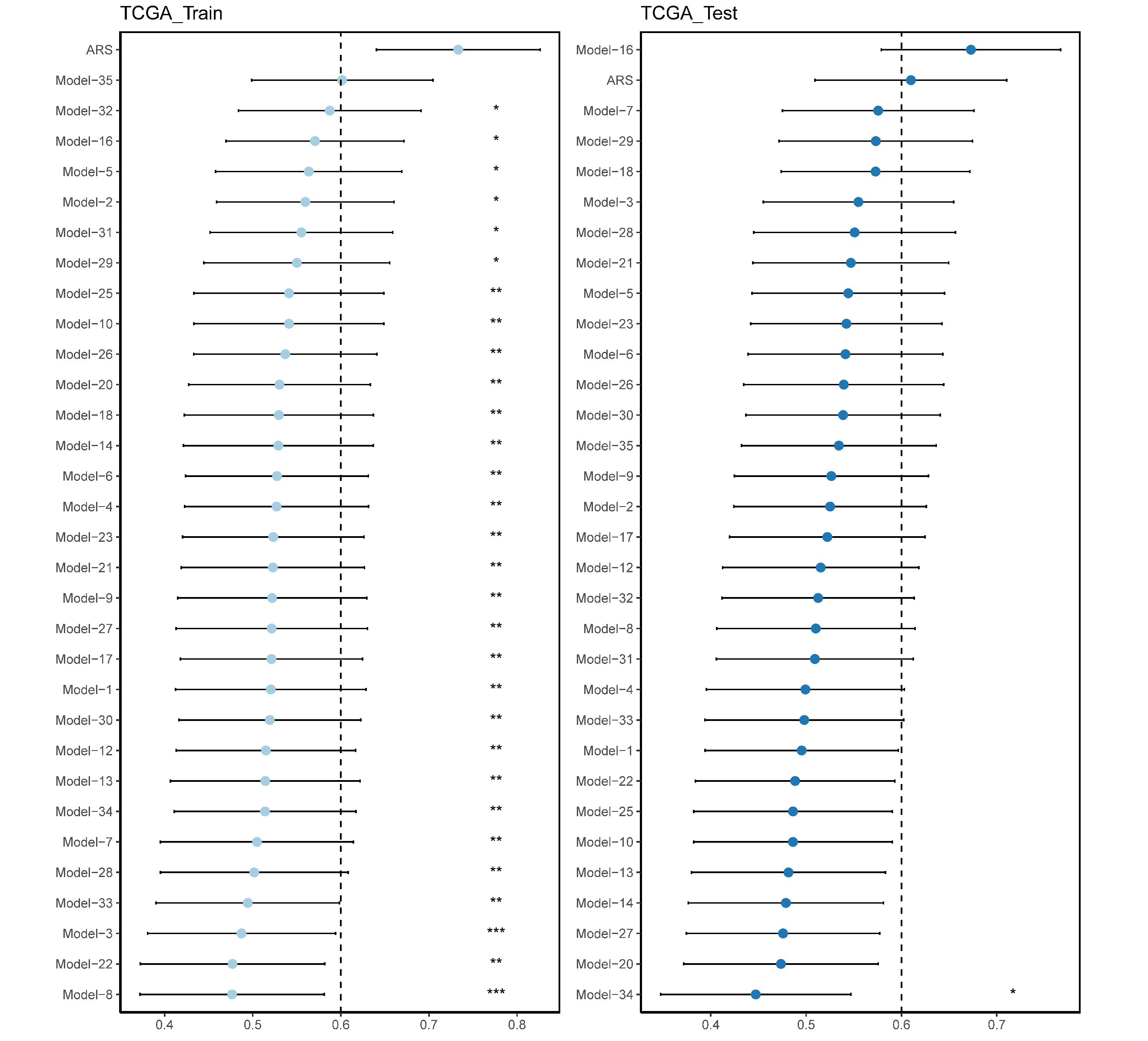


**Figure S6.** Comparison of gene expression-based prognostic signatures in STS.


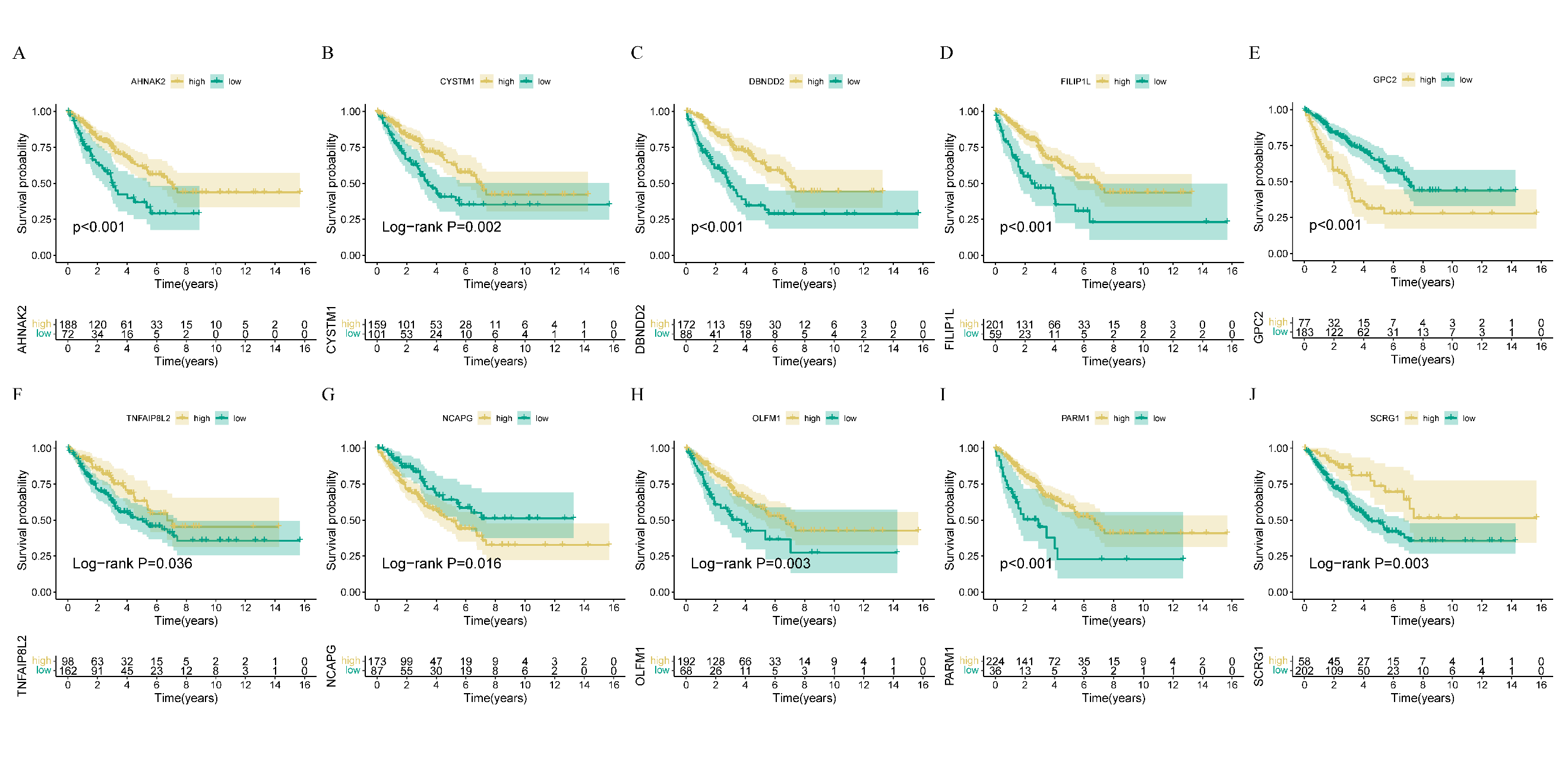


**Figure S7.** The KM survival plotter curve of the ten hub genes.

**
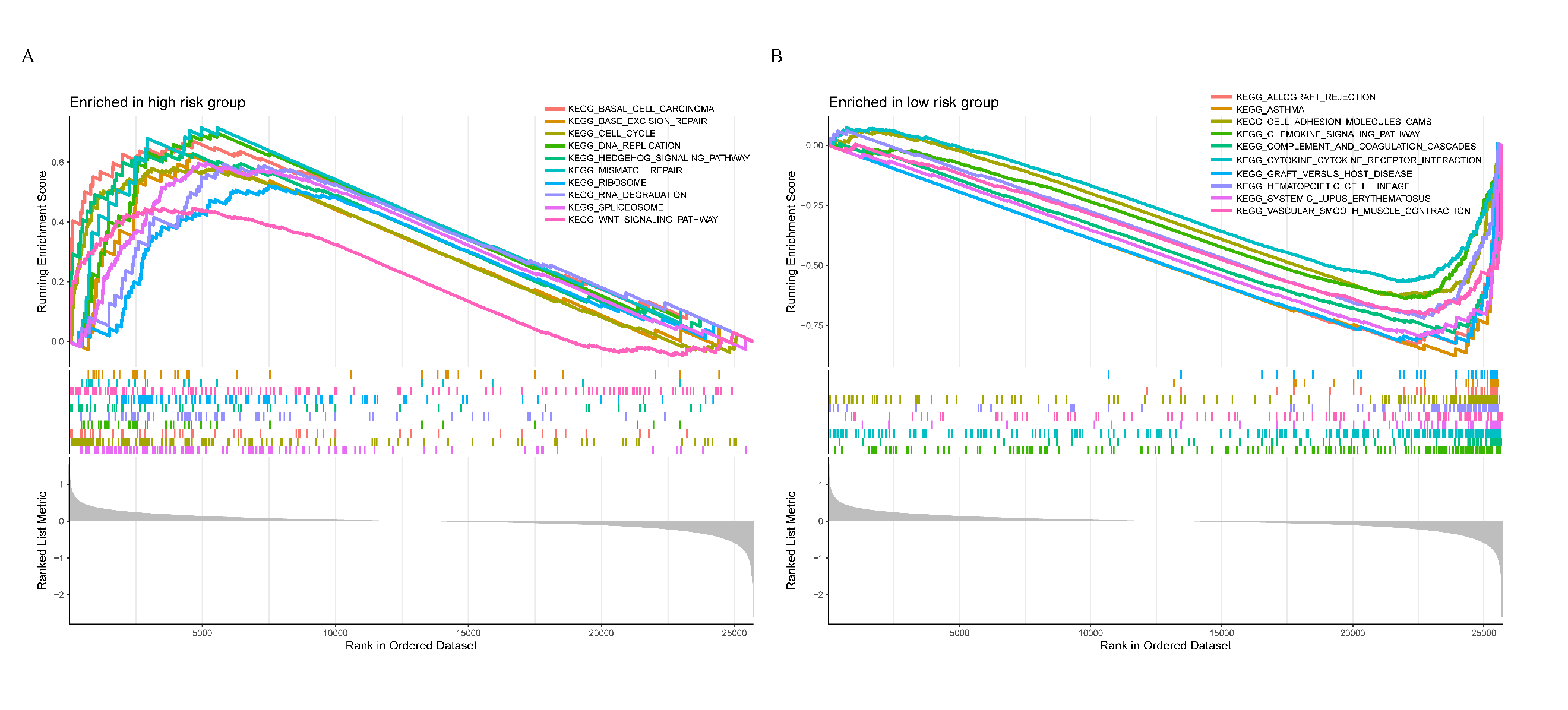
**

**Figure S8.** The GSEA enrichment results in the high- and low-risk groups.

**
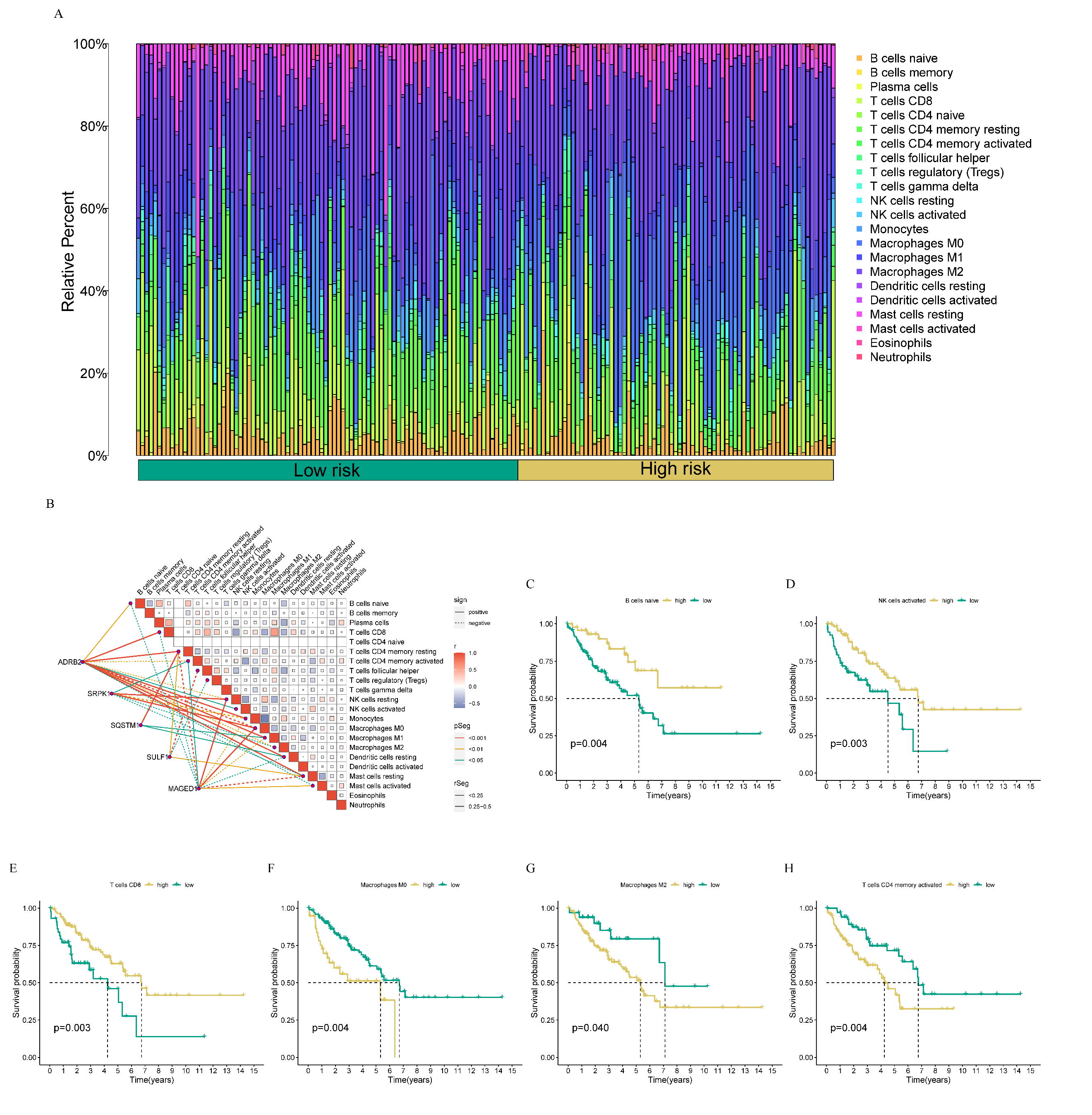
**

**Figure S9.** Correlation between the novel ARSig and immune cell infiltration. (A) The relative abundance of immune cells based on CIBERSORT in the different risk groups. (B) Correlation of signature ARGs with immune infiltration cells in STS. (C-H) The prognostic value of B cell naïve, NK cells activated, T cells CD8, Macrophage M0, Macrophage M2, and T cells CD4 memory activated.


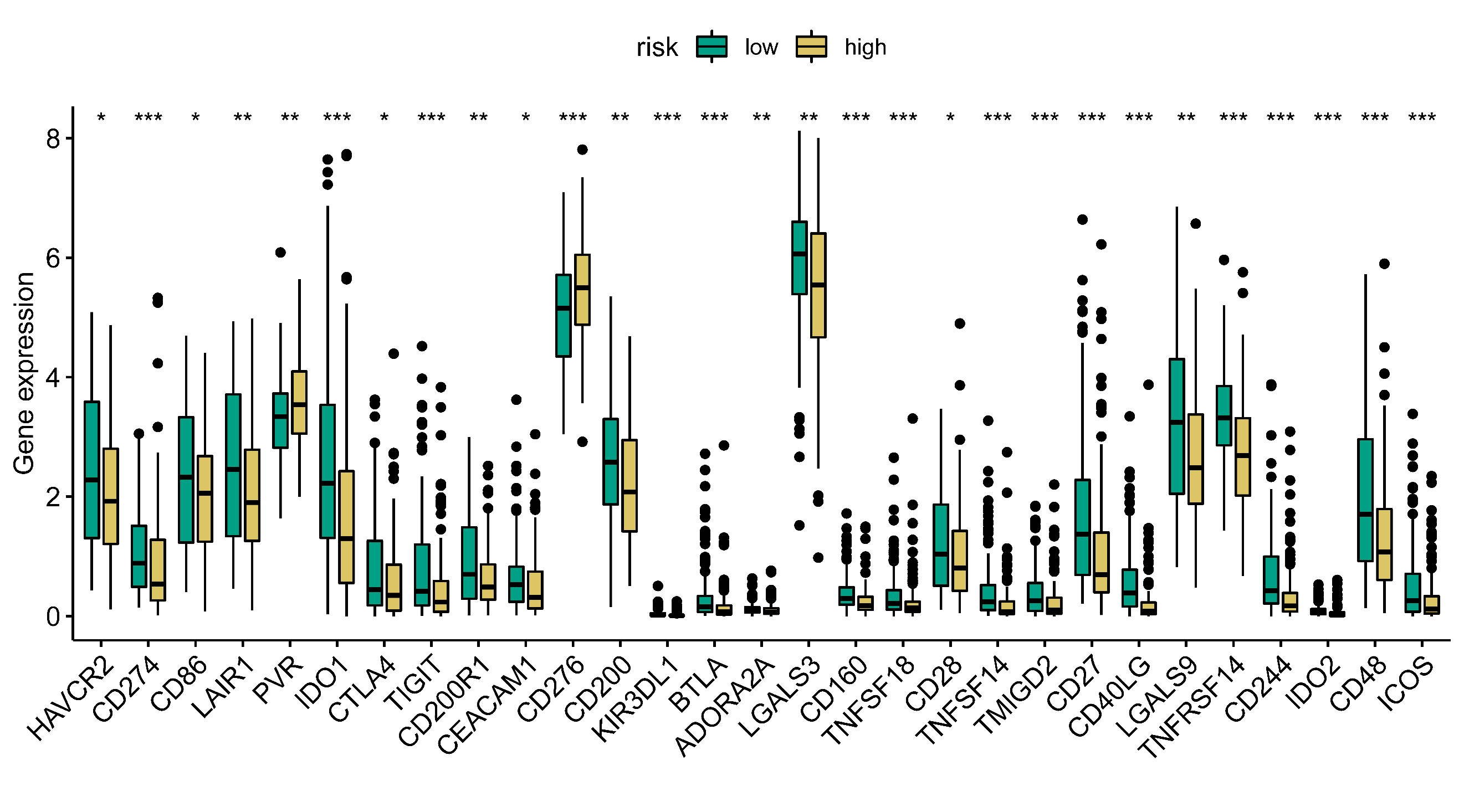


**Figure S10.** Differential expression levels of the immune checkpoint genes in the low-risk and high-risk groups.


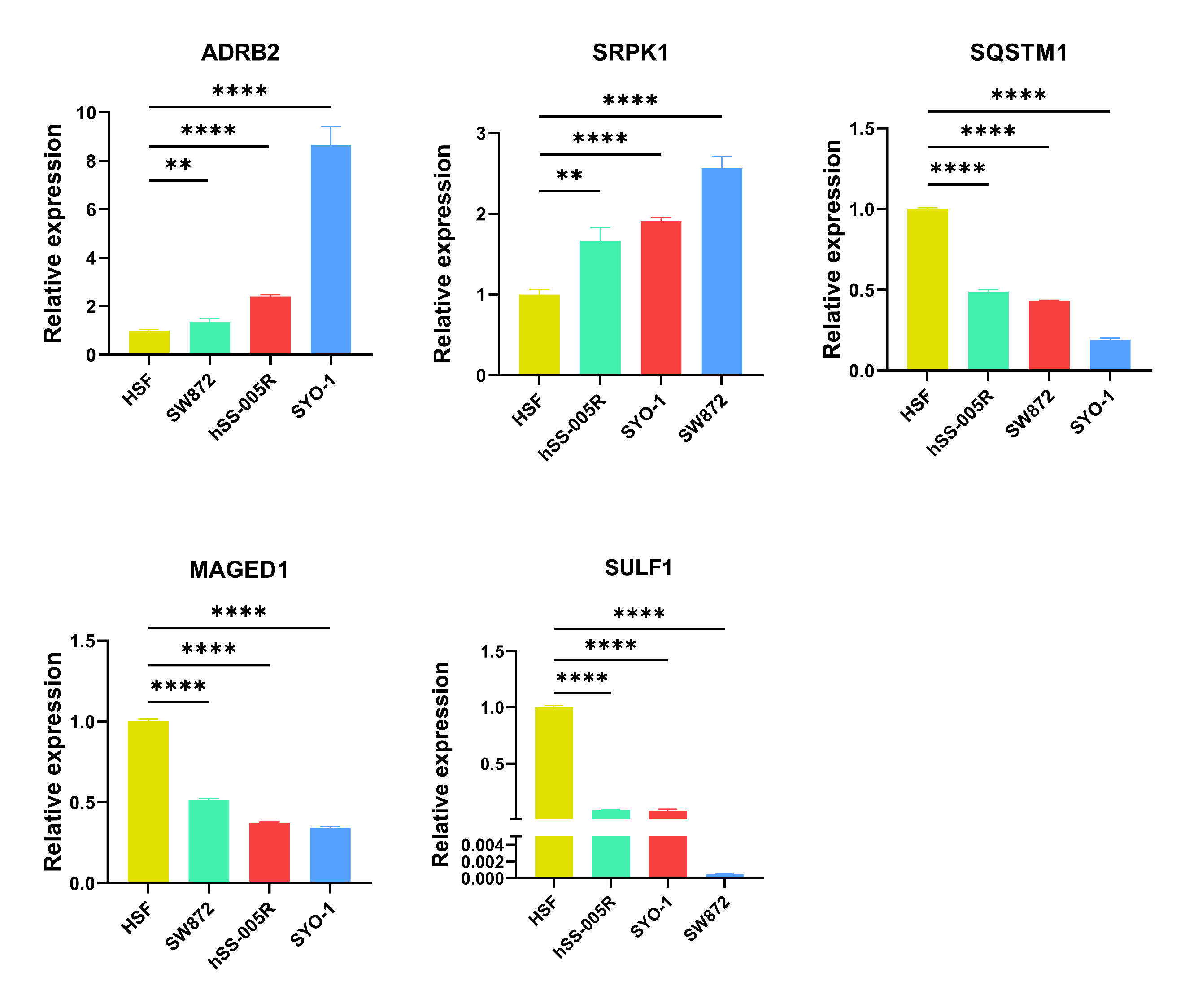


**Figure S11.** Verification of the expression of signature ARGs in STS cell lines. (A) ARDB2. (B) SRPK1. (C) SQSTM1. (D) MAGED1. (E) SULF1. *p < 0.05, **p < 0.01, ***p < 0.001, ****p < 0.0001.
